# Phenylalanine ^15^N enrichment likely indicates fungal‐derived organic nutrient acquisition in mycoheterotrophic plants across fungal guilds

**DOI:** 10.1111/nph.71154

**Published:** 2026-04-29

**Authors:** Kenji Suetsugu, Chikage Yoshimizu, Jun Matsubayashi, Ichiro Tayasu

**Affiliations:** ^1^ Department of Biology, Graduate School of Science Kobe University Kobe Hyogo 657‐8501 Japan; ^2^ Institute for Advanced Research Kobe University 1‐1 Rokkodai, Nada‐ku Kobe Hyogo 657‐8501 Japan; ^3^ Research Institute for Humanity and Nature Kita‐ku Kyoto 603‐8047 Japan; ^4^ Faculty of Marine Science and Technology Fukui Prefectural University 1‐1 Gakuen‐cho, Obama Fukui 917‐0003 Japan

**Keywords:** amino acid‐specific isotope analysis, common mycorrhizal networks, isotopic fractionation, mixotrophy, mycoheterotrophy, mycorrhizal fungi, orchids, soil food webs

## Abstract

- Beyond fully mycoheterotrophic plants, many green plants may also obtain carbon from fungal partners. However, bulk stable isotope analyses often lack sufficient resolution in arbuscular mycorrhizal and rhizoctonia‐associated orchid systems, limiting inference of fungal‐derived organic nutrient acquisition.
- We measured δ^15^N values of glutamic acid (δ^15^N_Glu_) and phenylalanine (δ^15^N_Phe_) in 10 fully mycoheterotrophic species spanning arbuscular mycorrhizal, ectomycorrhizal, and saprotrophic fungal guilds, as well as in three photosynthetic species with contrasting inferred nutritional modes, together with co‐occurring autotrophic references and, where available, fungal material. This Glu‐Phe framework was originally developed for animal food‐web studies, in which trophic position is estimated from strong ^15^N_Glu_ enrichment (*c.* 7–8‰ per trophic step) and minimal ^15^N_Phe_ enrichment (*c.* 0–1‰).
- We found that mycoheterotrophic plants invert this pattern, showing minimal ^15^N_Glu_ enrichment (−1.2 to 3.3‰) but pronounced ^15^N_Phe_ enrichment (11.2 to 19.6‰) relative to their fungal partners. They also exhibited amino acid δ^15^N profiles distinct from autotrophs, consistent with uptake of fungal‐derived organic nitrogen.
- To our knowledge, such strong ^15^N_Phe_ enrichment relative to nutrient sources has not been reported in other organisms. Overall, amino acid δ^15^N profiles complement bulk δ^2^H, δ^13^C, and δ^15^N values for assessing fungal‐derived organic nutrient acquisition in green plants.

## Introduction

Mycoheterotrophy represents an exceptional nutritional strategy among terrestrial plants (Merckx, 2013). Mycoheterotrophic plants acquire organic carbon from arbuscular mycorrhizal (AM) and ectomycorrhizal (ECM) fungi, as well as from saprotrophic (SAP) fungi that decompose organic matter (Bidartondo *et al*., 2004; Martos *et al*., 2009; Ogura‐Tsujita *et al*., 2009; Merckx *et al*., 2010; Lee *et al*., 2015; Gomes *et al*., 2020; Suetsugu *et al*., 2020). Once considered anomalies, these plants have recently attracted renewed attention because of their ecological significance (Merckx *et al*., 2024). Accumulating evidence indicates that many green plants may acquire carbon through mycorrhizal networks (Gebauer & Meyer, 2003; Selosse & Roy, 2009; Klein *et al*., 2016), although the extent and generality of such transfer, especially in AM systems, remain debated (Karst *et al*., 2023; Robinson *et al*., 2024). Determining the prevalence of partial mycoheterotrophy, or mixotrophy, in green plants is therefore central to understanding the ecological and evolutionary dynamics of mycorrhizal associations (Merckx *et al*., 2024).

Laboratory and *in situ* CO_2_ labeling experiments can directly demonstrate carbon transfer through common mycorrhizal networks (Simard *et al*., 1997; Klein *et al*., 2016). However, these approaches are labor‐intensive, technically demanding, and capture only short‐term fluxes (Hynson *et al*., 2013). They also struggle to disentangle mycorrhizal‐mediated transfer from alternative pathways such as soil respiration or root exudation (Karst *et al*., 2023; Robinson *et al*., 2024).

Consequently, stable isotope analysis (δ^2^H, δ^13^C, δ^15^N, and δ^18^O) has been widely used to infer the trophic modes of partially and fully mycoheterotrophic plants (Gebauer & Meyer, 2003; Hynson *et al*., 2013; Gebauer *et al*., 2016). This approach is based on the observation that fungi are typically enriched in ^2^H, ^13^C, and ^15^N relative to their substrates and to autotrophic plants, reflecting isotopic routing of plant‐derived photoassimilates and fractionation during heterotrophic metabolism and nitrogen transformations (Gebauer & Dietrich, 1993; Gleixner *et al*., 1993; Ziegler, 1995; Hobbie *et al*., 1999; Griffith, 2004). Among these elements, δ^13^C has served as a primary indicator of fungal carbon acquisition because it is directly informative for carbon source attribution (Selosse *et al*., 2025). Accordingly, partially mycoheterotrophic plants generally exhibit intermediate δ^13^C values consistent with mixed carbon sources (Gebauer & Meyer, 2003; Hynson *et al*., 2013; Gebauer *et al*., 2016).

However, detecting partial mycoheterotrophy from ^13^C enrichment alone is challenging in AM‐associated plants and rhizoctonia‐associated orchids, which together comprise nearly 90% of all plant species (Selosse *et al*., 2025). Their associated fungal groups often induce only modest ^13^C enrichment relative to typical C_3_ autotrophs, limiting the diagnostic value of bulk carbon isotope data (Courty *et al*., 2011; Zahn *et al*., 2023). Consequently, bulk ^2^H and ^15^N enrichment patterns have been proposed as complementary indicators because many fungal‐derived organic compounds transferred to plants, including amino acids, contain both nitrogen and hydrogen (Hynson *et al*., 2013; Gebauer *et al*., 2016). Nevertheless, ^2^H enrichment alone does not provide definitive evidence of partial mycoheterotrophy (Selosse *et al*., 2025), as species‐specific physiological processes can influence hydrogen fractionation even in autotrophs (e.g. Baan *et al*., 2025). Similarly, although many mycoheterotrophic plants show concurrent ^13^C and ^15^N enrichment, the magnitudes of these enrichments are often uncorrelated, likely reflecting variation among fungal species, fungal guilds, and plant physiology (Hynson *et al*., 2016; Gomes *et al*., 2020; Jacquemyn *et al*., 2021). Bulk ^15^N enrichment therefore has limited value as a standalone indicator of fungal dependence.

In this context, compound‐specific analysis of amino acid δ^15^N (δ^15^N_AAs_) may provide finer resolution of nutrient exchange between mycoheterotrophs and fungi. In food‐web studies developed primarily for animals, glutamic acid (Glu) is treated as a trophic amino acid because it typically becomes strongly enriched in ^15^N during trophic transfer, whereas phenylalanine (Phe) remains largely unchanged and serves as a source amino acid (Chikaraishi *et al*., 2009; McMahon & McCarthy, 2016; Ishikawa, 2018). This contrast enables estimation of trophic position (TP) across diverse ecosystems, including complex soil systems in which fungi mediate interactions (Steffan *et al*., 2015; Pollierer *et al*., 2019, 2020). TP denotes the number of steps above primary producers, *c*. 1 for producers, *c*. 2 for herbivores, and *c*. 3 for carnivores. It is commonly calculated as TP = [(δ^15^N_Glu_ − δ^15^N_Phe_ + *β*)/Δ] + 1, where Δ is the trophic discrimination factor (*c*. 7.6‰) and *β* is the baseline offset between Glu and Phe in primary producers (*c*. +8.4‰ for terrestrial C_3_ systems) (Chikaraishi *et al*., 2010).

Mycoheterotrophy can be conceptualized as a consumer–resource interaction because organic nutrients are obtained directly from fungal partners (Merckx, 2013). Bulk ^13^C and ^15^N enrichment in mycoheterotrophic plants relative to fungal partners often parallels enrichment patterns observed in consumers relative to their diet (Schiebold *et al*., 2017; Suetsugu *et al*., 2025; Suetsugu & Okada, 2025; but see also Gomes *et al*., 2023; Zahn *et al*., 2023). Anatomical, transcriptomic, and labeling studies further demonstrate that amino acids constitute major fungal‐derived nitrogen sources in many mycoheterotrophs, particularly orchids (Bougoure *et al*., 2010; Imhof *et al*., 2013; Fochi *et al*., 2017). Because fungi supplying nutrients to mycoheterotrophs usually occupy TPs above 2 (Steffan *et al*., 2015; Pollierer *et al*., 2020), mycoheterotrophs would be expected to exceed a TP of 3 if nitrogen assimilation followed patterns typical of fungivorous animals.

However, key physiological differences separate plants from animals. Plants generally have lower nitrogen concentrations and rely strongly on internal nitrogen recycling (Elser *et al*., 2000; Xu *et al*., 2012), so excretion‐driven processes responsible for trophic ^15^N enrichment in animals may be weak or absent. In addition, phenylalanine ammonia‐lyase (PAL) activity during lignin and phenylpropanoid biosynthesis deaminates phenylalanine and is expected to enrich residual Phe in ^15^N (Naito *et al*., 2016; Kendall *et al*., 2019). As a result, δ^15^N_AAs_ frameworks developed for animals are unlikely to apply directly to mycoheterotrophic plants. Nevertheless, because mycoheterotrophs obtain a substantial proportion of nitrogen from fungi in organic form (Gebauer & Meyer, 2003), they should display δ^15^N_AAs_ patterns distinct from those of autotrophs, which primarily assimilate inorganic nitrate and ammonium (Xu *et al*., 2012).

To date, no study has directly compared δ^15^N_Glu_ and δ^15^N_Phe_ patterns in mycoheterotrophic plants with those of their fungal partners, which represent direct nutrient sources, and co‐occurring autotrophs, which provide local reference baselines and approximate the ultimate carbon sources that support the fungi. The only previous δ^15^N_AAs_ study, which lacked local baselines, concluded that a fully mycoheterotrophic species, *Monotropastrum humile*, obtains nitrogen primarily from inorganic sources, based on a TP near 1 (Fan *et al*., 2025). However, its strong bulk ^15^N enrichment (+8.6‰ relative to autotrophs) is more consistent with uptake of fungal‐derived organic nitrogen (Suetsugu *et al*., 2020). Fan *et al*. (2025) also reported very high δ^15^N_Phe_ values (21.6‰) in *M. humile*, suggesting that mycoheterotrophy may promote ^15^N_Phe_ enrichment and yield deceptively low TP estimates. Moreover, because mycoheterotrophs, especially AM‐associated taxa, can also acquire inorganic nitrogen directly from soil through their roots (Gomes *et al*., 2020), broader comparative analyses of ^15^N_Glu_ and ^15^N_Phe_ enrichment patterns across mycoheterotrophic plants remain necessary.

We therefore examined ^15^N_Glu_ and ^15^N_Phe_ enrichment patterns across diverse mycoheterotrophic taxa associated with AM, ECM, and SAP fungi, relative to co‐occurring autotrophs and fungal partners. We also analyzed three photosynthetic species with contrasting nutritional modes: *Platanthera minor*, a partially mycoheterotrophic ECM orchid with high bulk ^15^N enrichment; *Goodyera velutina*, a rhizoctonia‐associated orchid that is largely autotrophic but likely acquires some fungal carbon; and *Trillium camschatcense*, a presumed autotrophic AM plant with elevated δ^13^C values (Yagame *et al*., 2012; Suetsugu *et al*., 2019; Murata‐Kato *et al*., 2022).

Using these datasets, we evaluated whether isotopic patterns are consistent with mycoheterotrophs obtaining nitrogen mainly as fungal‐derived organic compounds and therefore displaying δ^15^N_AAs_ patterns distinct from those of autotrophs. We further predicted guild‐specific differences, with AM‐associated mycoheterotrophs showing weaker ^15^N_Phe_ enrichment than ECM‐ or SAP‐associated taxa. Finally, we discuss the strengths and limitations of compound‐specific δ^15^N_AAs_ analysis for detecting fungal carbon acquisition in green plants.

## Materials and Methods

### Study species and sampling localities

Across nine sites (P1–P9) spanning warm‐temperate western Japan to cool‐temperate northern Japan, we collected 10 fully mycoheterotrophic species associated with three fungal guilds (AM, ECM, and SAP), together with co‐occurring autotrophic reference plants. We also sampled three photosynthetic species representing contrasting inferred nutritional modes, including a partially mycoheterotrophic orchid (*Platanthera minor* (Miq.) Rchb.f.), a rhizoctonia‐associated orchid with unresolved nutritional mode (*Goodyera velutina* Maxim. ex Regel), and a presumed autotrophic AM plant with elevated δ^13^C values (*Trillium camschatcense* Ker Gawl.) (Table 1; Figs S1, 1). Because *Lecanorchis kiusiana* Tuyama produces lignified stalks that persist into the following year, we sampled lignin‐poor inflorescences at the emerging stage and lignin‐rich persistent infructescences to evaluate the effect of lignification on δ^15^N_Phe_ (Kendall *et al*., 2019).

**Table 1:** Summary of focal plant species included in the study, with family, nutritional mode, and fungal partner guild.

Site	Species	Family	Nutritional mode	Fungal partner guild
P1	*Burmannia championii*	Burmanniaceae	FMH	AM
P1	*Sciaphila japonica*	Triuridaceae	FMH	AM
P2	*Sciaphila tosaensis*	Triuridaceae	FMH	AM
P3	*Burmannia cryptopetala*	Burmanniaceae	FMH	AM
P3	*Monotropa uniflora*	Ericaceae	FMH	ECM
P4	*Petrosavia sakuraii*	Petrosaviaceae	FMH	AM
P5	*Lecanorchis kiusiana*	Orchidaceae	FMH	ECM
P5	*Gastrodia nipponica*	Orchidaceae	FMH	SAP
P5	*Platanthera minor*	Orchidaceae	PMH	ECM
P6	*Burmannia championii*	Burmanniaceae	FMH	AM
P6	*Goodyera velutina*	Orchidaceae	IMH/PMH	SAP/endophytic
P7	*Gastrodia pubilabiata*	Orchidaceae	FMH	SAP
P8	*Cremastra aphylla*	Orchidaceae	FMH	SAP
P8	*Trillium camschatcense*	Melanthiaceae	Presumed autotroph	AM
P9	*Trillium camschatcense*	Melanthiaceae	Presumed autotroph	AM

Because *Goodyera velutina* is associated with rhizoctonia fungi and shows no clear ^13^C signal, its nutritional mode and fungal partner guild are not yet fully resolved. AM, arbuscular mycorrhizal; ECM, ectomycorrhizal; FMH, full mycoheterotroph; IMH, initial mycoheterotroph; PMH, partial mycoheterotroph; SAP, saprotrophic.

**Fig. 1:**
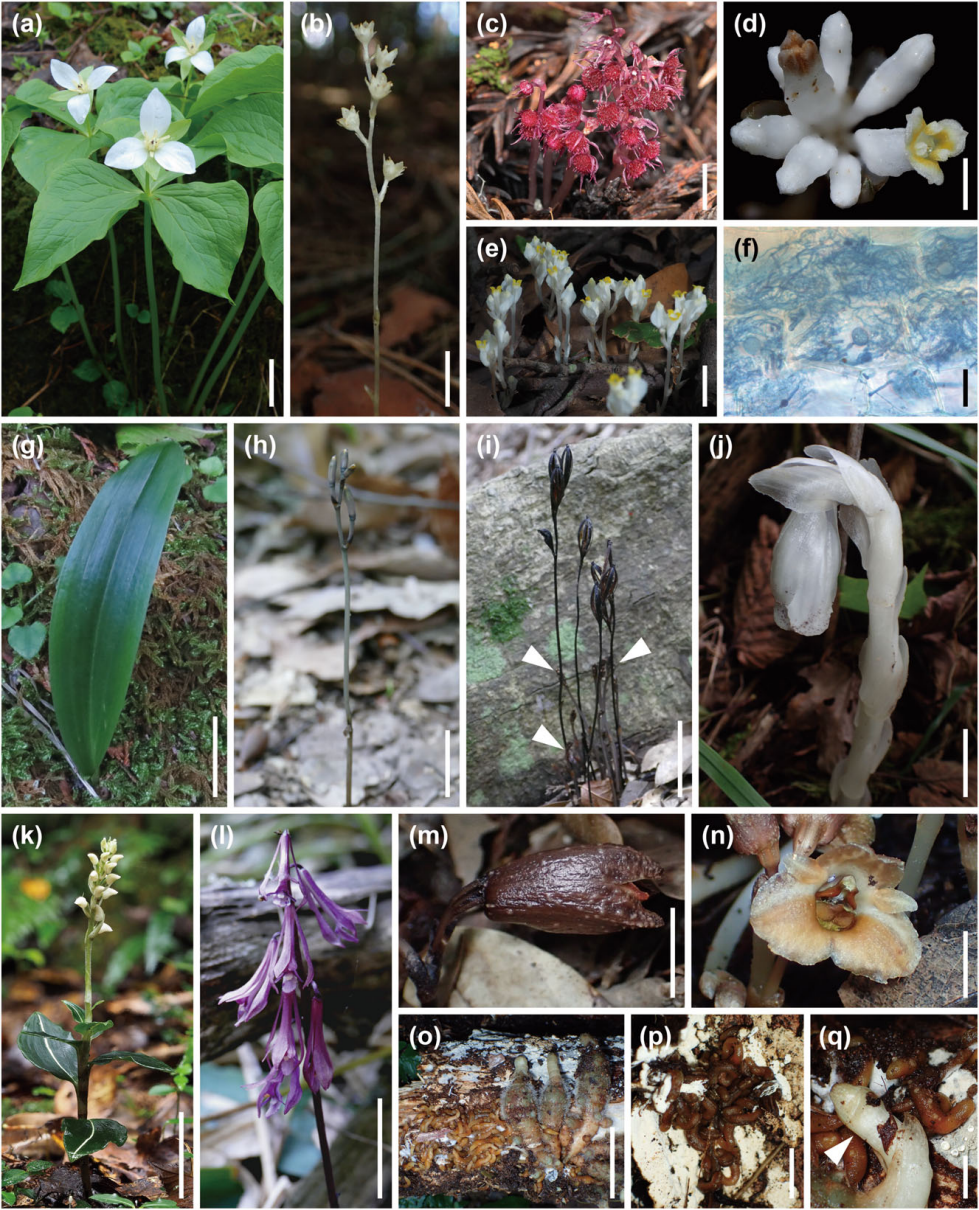
Representative target species analyzed in this study. (a) A presumed autotrophic plant, *Trillium camschatcense*, associated with arbuscular mycorrhizal fungi and exhibiting ^13^C enrichment. (b–f) Fully mycoheterotrophic plants associated with arbuscular mycorrhizal fungi: *Petrosavia sakuraii* (b), *Sciaphila tosaensis* (c), *Burmannia championii* (d), *Burmannia cryptopetala* (e), and a longitudinal root section of *B. cryptopetala* showing arbuscular mycorrhizal hyphal coils (f). (g) *Platanthera minor*, a partially mycoheterotrophic orchid associated with ectomycorrhizal fungi. (h–j) Fully mycoheterotrophic species associated with ectomycorrhizal fungi: *Lecanorchis kiusiana* at the emerging stage (h) and fruiting stage (i), and *Monotropa uniflora* (j). Note that in (i), emerging individuals of *L. kiusiana* are visible at the base of a fruiting individual (arrows). (k) *Goodyera velutina*, a rhizoctonia‐associated orchid. (l–q) Fully mycoheterotrophic plants associated with saprotrophic, non‐rhizoctonia fungi: *Cremastra aphylla* (l), *Gastrodia nipponica* (m), *Gastrodia pubilabiata* (n), *G. pubilabiata* with its fungal partner *Resinicium* sp. on deadwood of *Cryptomeria japonica* (o), branched mycorrhizal roots of *G. pubilabiata* adhering to *Resinicium* sp. (p), and a close‐up of mycorrhizal roots and scape of *G. pubilabiata* adhering to *Resinicium* sp., with an arrow indicating the scape (q). Bars: (a, k, l) 5 cm; (b, c, e, j, m, p) 1 cm; (n, q) 5 mm; (d) 2 mm; (f) 50 μm; (g–i) 2 cm; and (o) 3 cm.

Co‐occurring autotrophic reference species, representing indirect carbon sources, were sampled within 2 m × 2 m quadrats containing target plants. At sites P1, P5, and P9, samples were taken within a 5 m radius because reference plants were scarce. Although this broader radius may have increased baseline variance because of light heterogeneity, it was unavoidable given the limited availability of suitable reference plants. Reference plants were collected mainly from the understory at heights similar to those of mycoheterotrophs (5–30 cm aboveground), following established protocols (Gebauer & Meyer, 2003; Preiss & Gebauer, 2008). Because shaded understory foliage typically exhibits more depleted δ^13^C values than canopy‐derived carbon commonly transferred to mycorrhizal fungi (Courty *et al*., 2011), we also sampled leaves from canopy trees above 2 m, while recognizing that reachable branches may still underrepresent full‐crown gradients (Gebauer & Schulze, 1991). Leaves were collected from photosynthetic species, including partially mycoheterotrophic species, whereas leafless, fully mycoheterotrophic plants were represented by floral stalks.

Where possible, we also sampled fungal partners to characterize the isotopic values of direct nutrient sources. We collected fruiting bodies of the saprotroph *Resinicium* sp. and samples of decayed *Cryptomeria japonica* (L.f.) D. Don wood colonized by *Resinicium* sp. and indirectly supporting *Gastrodia pubilabiata* Y. Sawa, allowing comparisons among substrate wood, fungus, and plant. Because AM fungi lack visible fruiting bodies, obtaining pure fungal material was extremely difficult for AM associations, particularly in amounts sufficient for amino acid isotope analyses. Accordingly, for the AM‐associated *Burmannia cryptopetala* Makino, we compared root segments containing abundant fungal coils with fungus‐free floral stalks. Roots of *B. cryptopetala* are translucent white, with darkened regions indicating dense fungal coils, and these regions were selected for analysis. A summary of focal species, nutritional category, fungal partner type, and sampled material is provided in Table 1, with more detailed information given in Tables S1 and S2.

### Bulk δ^13^C and δ^15^N analyses

Carbon and nitrogen stable isotope ratios were measured using a Delta V Advantage mass spectrometer (Thermo Fisher Scientific, Waltham, MA, USA) coupled to a Flash EA 1112 elemental analyzer via a ConFlo IV interface (Thermo Fisher Scientific) at the Research Institute for Humanity and Nature (Kyoto, Japan). Relative isotope abundances were calculated as δ^13^C or δ^15^N = (*R*_sample_/*R*_standard_ − 1) × 1000 [‰], where *R*_sample_ represents the ^13^C/^12^C or ^15^N/^14^N ratio in the sample, and *R*_standard_ represents the corresponding ratio for Vienna Pee Dee Belemnite or atmospheric N_2_, respectively.

Calibration was performed using three laboratory standards, CERKU‐01 (for N), CERKU‐02 (for C and N), and CERKU‐03 (for C), all traceable to international standards (Tayasu *et al*., 2011). Analytical SDs of δ^13^C and δ^15^N were < 0.08‰ and 0.15‰, respectively. Total C and N concentrations were calculated from sample weights and gas concentrations (CO_2_ and N_2_) calibrated with laboratory standards (Tayasu *et al*., 2011).

### δ^15^N_Glu_ and δ^15^N_Phe_ analyses

Compound‐specific δ^15^N analyses of amino acids were performed on a subset of specimens selected to represent each focal taxon, where biomass permitted, while limiting analytical effort. We prioritized samples that: (1) represented each focal species at each site where sufficient biomass was available; (2) included contrasting tissues expected to differ in amino acid routing; and (3) included co‐occurring autotrophic reference plants used to calculate enrichment factors. Samples were prepared following the derivatization procedures of Chikaraishi *et al*. (2015) with minor modifications. Before N‐pivaloyl/isopropyl derivatization, samples were purified by cation‐exchange chromatography (Takano *et al*., 2010).

δ^15^N_AAs_ values were determined by gas chromatography/combustion/isotope‐ratio mass spectrometry using a TRACE GC Ultra instrument (Thermo Fisher Scientific) coupled to a Delta V Advantage via GC IsoLink and ConFlo IV interfaces (Thermo Fisher Scientific). Reference mixtures of five amino acids (leucine, norleucine, aspartic acid, phenylalanine, and hydroxyproline) with known δ^15^N values (−9.17 to +35.20‰; Shoko Science Co., Kanagawa, Japan) were analyzed alongside samples. Analytical SDs for the reference standards were < 0.7‰.

### Calculation and statistics

For each site, enrichment factors (ε^13^C, ε^15^N, ε^15^N_Glu_, and ε^15^N_Phe_) were calculated as ε = δ_S_ − δ_REF_, where δ_S_ is the isotope value of a target specimen, and δ_REF_ is the mean isotope value of autotrophic reference plants in the same plot (Preiss & Gebauer, 2008). For bulk isotopes, understory leaves were used as plot‐level autotrophic baselines (Gebauer & Meyer, 2003; Preiss & Gebauer, 2008). For δ^15^N_Glu_ and δ^15^N_Phe_, enrichment factors were calculated without separating understory and canopy leaves because δ^15^N_AAs_ values were not expected to differ systematically between strata.

Group differences in isotopic metrics and carbon and nitrogen concentrations among mycoheterotrophic plants and their potential carbon sources were assessed using linear models, followed by Tukey–Kramer *post hoc* tests, and effect sizes were calculated as Cohen's *d*. When any group had *n* ≤ 2, no statistical tests were performed, and the results are described as trends rather than as statistically significant differences. All statistical analyses were conducted in R (R Core Team, 2025).

TP was calculated using the conventional Glu‐Phe equation (Chikaraishi *et al*., 2010): TP = (δ^15^N_Glu_ − δ^15^N_Phe_ + 8.4)/7.6 + 1. Because this framework was developed primarily for animals, the resulting values were treated as descriptive indices rather than as literal TPs. Direct measurements of fungal δ^15^N_Glu_ or δ^15^N_Phe_ were available only for *Resinicium* sp. associated with *G. pubilabiata*. We also measured δ^15^N_Glu_ and δ^15^N_Phe_ in the mycorrhizal root of *B. cryptopetala*. Because this tissue reflects a plant–fungus composite signal, it was treated as an approximate proxy for an AM fungal source rather than as a direct fungal measurement (Tables S1, S2). In other cases, fungal δ^15^N_Glu_ and δ^15^N_Phe_ values were estimated from co‐occurring autotrophic plants using published offsets between fungi and their carbon sources (*c*. +6.5‰ for δ^15^N_Glu_ and −0.7‰ for δ^15^N_Phe_; Steffan *et al*., 2015).

## Results

### Bulk δ^13^C and δ^15^N analyses

**Fig. 2:**
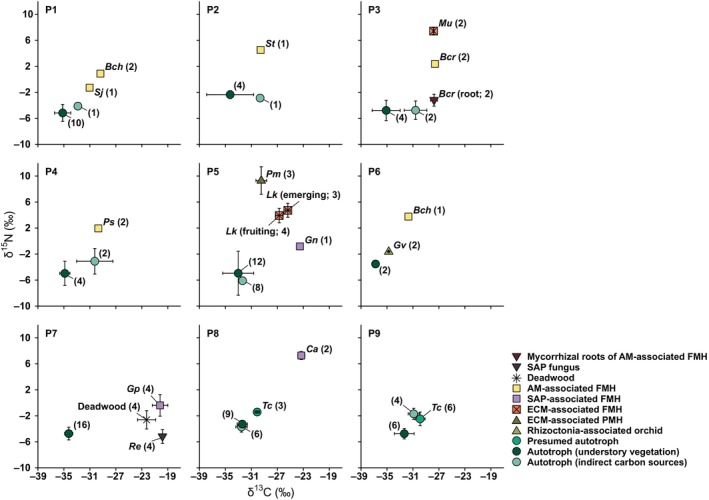
Mean (±SD) δ^13^C and δ^15^N values of fully mycoheterotrophic plants associated with arbuscular mycorrhizal (AM), ectomycorrhizal (ECM), and saprotrophic (SAP) fungi, a partially mycoheterotrophic plant associated with ECM, a rhizoctonia‐associated orchid, a presumed autotrophic plant with ^13^C enrichment, and their neighboring autotrophic plants, deadwood, and fungal partners at each study site (P1–P9). PMH, partial mycoheterotroph; FMH, full mycoheterotroph. *Bch*, *Burmannia championii*; *Bcr*, *Burmannia cryptopetala*; *Ca*, *Cremastra aphylla*; *Gn*, *Gastrodia nipponica*; *Gp*, *Gastrodia pubilabiata*; *Gv*, *Goodyera velutina*; *Lk*, *Lecanorchis kiusiana*; *Mu*, *Monotropa uniflora*; *Pm*, *Platanthera minor*; *Ps*, *Petrosavia sakuraii*; *Re*, *Resinicium* sp.; *Sj*, *Sciaphila japonica*; *St*, *Sciaphila tosaensis*; *Tc*, *Trillium camschatcense*. Numbers in parentheses indicate sample size.

Fully mycoheterotrophic plants had δ^13^C values (−26.2 ± 3.5‰; *n* = 25) that were significantly higher than those of co‐occurring autotrophic understory species (−33.9 ± 1.9‰; mean ± SD; *n* = 67; Cohen's *d* = 3.1; *P* < 0.001) and canopy trees (−31.7 ± 1.3‰; *n* = 24; Cohen's *d* = 2.1; *P* < 0.001; Fig. 2; Table S1). Among fully mycoheterotrophic species, variation in ^13^C enrichment partly corresponded to differences in their associated fungal guilds. Fully mycoheterotrophic plants associated with SAP fungi (−21.6 ± 1.9‰; *n* = 7) exhibited significantly higher δ^13^C values than those associated with ECM fungi (−26.5 ± 1.0‰; *n* = 9; Cohen's *d* = 3.4) and AM fungi (−29.5 ± 1.4‰; *n* = 9; Cohen's *d* = 4.9; *P* < 0.001).

Similarly, fully mycoheterotrophic plants showed δ^15^N values (3.0 ± 2.9‰; *n* = 25) that were significantly higher than those of co‐occurring autotrophic understory species (−4.5 ± 1.8‰; *n* = 67; Cohen's *d* = 3.5; *P* < 0.001) and canopy trees (−4.2 ± 1.8‰; *n* = 24; Cohen's *d* = 3.0; *P* < 0.001). Among fully mycoheterotrophic plants, ECM‐associated taxa (5.0 ± 1.7‰; *n* = 9) had higher δ^15^N values than SAP‐associated taxa (1.7 ± 4.0‰; *n* = 7; Cohen's *d* = 1.1; *P* < 0.05) and AM‐associated taxa (1.9 ± 1.7‰; *n* = 9; Cohen's *d* = 1.8; *P* < 0.05). The partially mycoheterotrophic *P. minor* (ECM‐associated) had higher δ^15^N values (9.3 ± 2.1‰; *n* = 3) than co‐occurring *L. kiusiana* (4.3 ± 1.1‰; *n* = 7; Cohen's *d* = 3.6; *P* < 0.05) and *G. nipponica* (−0.8‰; *n* = 1). Its δ^13^C values (−29.5 ± 0.8‰; *n* = 3) were lower than those of *L. kiusiana* (−26.2 ± 0.8‰; *n* = 7; Cohen's *d* = −4.0; *P* < 0.05) and *G. nipponica* (−23.5‰; *n* = 1). The rhizoctonia‐associated orchid *G. velutina* appeared to have slightly higher δ^13^C and δ^15^N values (−34.7 ± 0.0‰ and −1.6 ± 0.1‰; *n* = 2) than co‐occurring autotrophs (−36.8 ± 0.5‰ and −3.5 ± 0.3‰; *n* = 2; Fig. 2).

*Trillium camschatcense*, a *Paris*‐type AM plant, showed significantly elevated δ^13^C and δ^15^N values (−30.0 ± 0.6‰ and − 2.1 ± 1.0‰; *n* = 9) relative to nearby autotrophic understory species (δ^13^C = −32.4 ± 1.1‰; Cohen's *d* = 2.6; *P* < 0.001 and δ^15^N = −3.9 ± 1.0‰; Cohen's *d* = 1.8; *P* < 0.005). No significant difference in δ^13^C values was found between *G. pubilabiata* (−20.2 ± 1.2‰; *n* = 4) and its fungal partner *Resinicium* sp. (−19.8 ± 0.3‰; *n* = 4; Cohen's *d* = −0.4; *P* = 0.92). By contrast, *G. pubilabiata* (−0.4 ± 1.7‰; *n* = 4) had significantly higher δ^15^N values than *Resinicium* sp. (−5.2 ± 1.1‰; *n* = 4; Cohen's *d* = 3.4; *P* < 0.01; Fig. 2).

Fully mycoheterotrophic plants had nitrogen concentrations (3.2 ± 1.1 mmol g^−1^; *n* = 25) that were significantly higher than those of co‐occurring autotrophic understory species (2.3 ± 0.8 mmol g^−1^; *n* = 67; Cohen's *d* = 1.0; *P* < 0.001) and canopy trees (1.7 ± 0.9 mmol g^−1^; *n* = 24; Cohen's *d* = 1.4; *P* < 0.001). Among fully mycoheterotrophic plants, nitrogen concentrations were similar across fungal guilds, with values of 3.1 ± 1.4 mmol g^−1^ in SAP‐associated taxa (*n* = 7), 3.2 ± 1.2 mmol g^−1^ in ECM‐associated taxa (*n* = 9), and 3.2 ± 0.8 mmol g^−1^ in AM‐associated taxa (*n* = 9). The partially mycoheterotrophic *P. minor* (ECM‐associated) showed nitrogen concentrations (3.2 ± 0.4 mmol g^−1^; *n* = 3) similar to those of *L. kiusiana* (3.3 ± 1.4 mmol g^−1^; *n* = 7; Cohen's *d* = −0.1; *P* = 0.87). The rhizoctonia‐associated orchid *G. velutina* appeared to have lower nitrogen concentrations (2.0 ± 0.3 mmol g^−1^; *n* = 2) than understory autotrophs (2.7 ± 0.0 mmol g^−1^; *n* = 2). *Trillium camschatcense* had nitrogen concentrations (2.6 ± 0.2 mmol g^−1^; *n* = 9) similar to those of autotrophic understory species (2.5 ± 0.5 mmol g^−1^; *n* = 15; Cohen's *d* = 0.3; *P* = 0.88) and canopy trees (2.5 ± 0.6 mmol g^−1^; *n* = 10; Cohen's *d* = 0.2; *P* = 0.98).

### δ^15^N_Glu_ and δ^15^N_Phe_ analyses

**Fig. 3:**
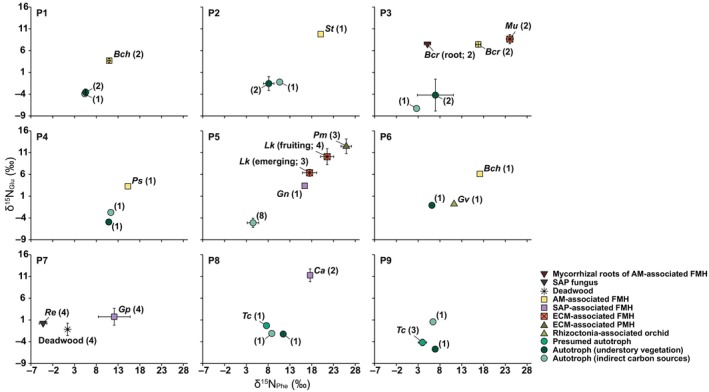
Mean (±SD) δ^15^N_Phe_ and δ^15^N_Glu_ values of fully mycoheterotrophic plants associated with arbuscular mycorrhizal (AM), ectomycorrhizal (ECM), and saprotrophic (SAP) fungi, a partially mycoheterotrophic plant associated with ECM, a rhizoctonia‐associated orchid, a presumed autotrophic plant with ^13^C enrichment, and their neighboring autotrophic plants, deadwood, and fungal partners at each study site (P1–P9). AM, arbuscular mycorrhizal; ECM, ectomycorrhizal; SAP, saprotrophic; PMH, partial mycoheterotroph; FMH, full mycoheterotroph. *Bch*, *Burmannia championii*; *Bcr*, *Burmannia cryptopetala*; *Ca*, *Cremastra aphylla*; *Gn*, *Gastrodia nipponica*; *Gp*, *Gastrodia pubilabiata*; *Gv*, *Goodyera velutina*; *Lk*, *Lecanorchis kiusiana*; *Mu*, *Monotropa uniflora*; *Pm*, *Platanthera minor*; *Ps*, *Petrosavia sakuraii*; *Re*, *Resinicium* sp.; *St*, *Sciaphila tosaensis*; *Tc*, *Trillium camschatcense*. Numbers in parentheses indicate sample size.

Fully mycoheterotrophic plants consistently showed significantly higher δ^15^N_Glu_ and δ^15^N_Phe_ values (δ^15^N_Glu_ = 6.6 ± 3.5‰ and δ^15^N_Phe_ = 17.2 ± 4.4‰; *n* = 23 for each) than co‐occurring autotrophic species (δ^15^N_Glu_ = −3.8 ± 2.1‰; Cohen's *d* = 3.6; *P* < 0.001 and δ^15^N_Phe_ = 6.6 ± 2.8‰; Cohen's *d* = 2.9; *P* < 0.001; *n* = 24; Fig. 3; Table S1).

**Fig. 4:**
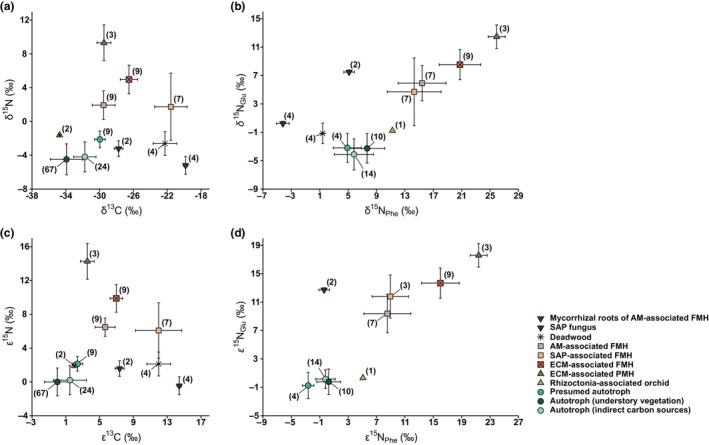
Mean (±SD) δ^13^C and δ^15^N values (a), δ^15^N_Phe_ and δ^15^N_Glu_ values (b), ε^13^C and ε^15^N values (c), and ε^15^N_Phe_ and ε^15^N_Glu_ values (d) of fully mycoheterotrophic plants associated with arbuscular mycorrhizal (AM), ectomycorrhizal (ECM), and saprotrophic (SAP) fungi, a partially mycoheterotrophic plant associated with ECM (*Platanthera minor*), a rhizoctonia‐associated orchid (*Goodyera velutina*), a presumed autotrophic plant with ^13^C enrichment (*Trillium camschatcense*), and their neighboring autotrophic plants, deadwood, and fungal partners. FMH, full mycoheterotroph; PMH, partial mycoheterotroph. Data are combined across nine study sites. Numbers in parentheses indicate sample size.

The extent of enrichment relative to co‐occurring autotrophic plants varied among mycoheterotrophic species, partly depending on their associated fungal guilds. ECM‐associated fully mycoheterotrophic plants showed the highest ε^15^N_Phe_ values (16.0 ± 2.7‰; *n* = 9), significantly exceeding those of SAP‐associated taxa (8.9 ± 2.6‰; *n* = 3; Cohen's *d* = 2.7; *P* = 0.005) and AM‐associated taxa (8.5 ± 3.3‰; *n* = 7; Cohen's *d* = 2.5; *P* < 0.01). For ε^15^N_Glu_, ECM‐associated taxa (13.6 ± 2.1‰; *n* = 9) were higher than AM‐associated taxa (9.4 ± 2.7‰; *n* = 7; Cohen's *d* = 1.8; *P* = 0.008), whereas the difference from SAP‐associated taxa (11.7 ± 3.0‰; *n* = 3; Cohen's *d* = 0.8; *P* = 0.5) was not significant (Fig. 4; Table S1). At the species level, ε^15^N_Glu_ ranged from 7.1‰ in *P. sakuraii* (*n* = 1) to 15.2 ± 1.8‰ in *L. kiusiana* at the fruiting stage (*n* = 4), whereas ε^15^N_Phe_ ranged from 4.2‰ in *P. sakuraii* (*n* = 1) to 17.0 ± 1.5‰ in *L. kiusiana* at the fruiting stage (*n* = 4). Within *L. kiusiana*, fruiting individuals had higher δ^15^N_Glu_ and δ^15^N_Phe_ values (10.1 ± 1.8‰ and 21.5 ± 1.5‰; *n* = 4) than emerging individuals (6.4 ± 0.8‰; Cohen's *d* = 2.4; *P* < 0.05 and 17.5 ± 1.6‰; Cohen's *d* = 2.6; *P* < 0.05; *n* = 3; Table S1).

The ECM‐associated partially mycoheterotroph *P. minor* exhibited even higher ε^15^N_Glu_ and ε^15^N_Phe_ values (17.6 ± 1.7‰ and 21.4 ± 1.2‰; *n* = 3) than co‐occurring *L. kiusiana* (13.6 ± 2.4‰; Cohen's *d* = 1.8; *P* < 0.05 and 15.3 ± 2.6‰; Cohen's *d* = 2.6; *P* < 0.01; *n* = 7) and *G. nipponica* (8.5‰ and 11.9‰; *n* = 1). The rhizoctonia‐associated *G. velutina* showed virtually no ^15^N_Glu_ enrichment but slight ^15^N_Phe_ enrichment (ε^15^N_Glu_ = 0.3‰ and ε^15^N_Phe =_ 5.0‰; *n* = 1) relative to a co‐occurring autotrophic reference (*n* = 1). By contrast, *T. camschatcense* showed no positive enrichment in Glu and significant depletion in Phe relative to autotrophic references (ε^15^N_Glu_ = −0.7 ± 1.8‰; Cohen's *d* = −0.3; *P* = 0.88 and ε^15^N_Phe_ = −2.6 ± 0.8‰; Cohen's *d* = −2.8; *P* = 0.01; *n* = 4).

The combination of weak ^15^N_Glu_ enrichment and strong ^15^N_Phe_ enrichment likely yields deceptively low TP estimates in mycoheterotrophs (0.9 ± 0.2, 0.5 ± 0.3, and 0.8 ± 0.4 for AM‐, ECM‐, and SAP‐associated taxa, respectively; Table S1). Notably, because of pronounced ^15^N_Phe_ enrichment (16.3 ± 3.7‰; range 11.2 to 19.6‰; *n* = 4) coupled with weak ^15^N_Glu_ enrichment (1.5 ± 1.9‰; range −1.2 to 3.3‰; *n* = 4) relative to fungal partners, *G. pubilabiata* had a TP index of 0.7 ± 0.2 (*n* = 4), whereas *Resinicium* sp. had a value of 2.7 ± 0.1 (*n* = 4; Table S1). Likewise, roots of *B. cryptopetala* containing abundant fungal coils had a TP index of 2.4 ± 0.1 (*n* = 2), whereas fungus‐free floral stalks had a value of 0.9 ± 0.0 (*n* = 2), reflecting no ^15^N_Glu_ enrichment (−0.1 ± 0.6‰; range −0.5 to 0.4‰; *n* = 2) and strong ^15^N_Phe_ enrichment (11.7 ± 0.5‰; range 11.3 to 12.0‰; *n* = 2) in stalks relative to roots. Estimated enrichment of mycoheterotrophic plants relative to fungi was 2.9 ± 2.7‰ for ^15^N_Glu_ and 9.3 ± 3.3‰ for ^15^N_Phe_ in AM‐associated taxa (*n* = 7); 7.2 ± 2.1‰ and 16.7 ± 2.7‰, respectively, in ECM‐associated taxa (*n* = 9); 11.1 ± 1.7‰ and 22.1 ± 1.2‰, respectively, in the partially mycoheterotrophic *P. minor* (*n* = 3); and 5.3 ± 2.0‰ and 9.7 ± 2.6‰, respectively, in SAP‐associated taxa (*n* = 3).

## Discussion

### ^15^N_Phe_ and ^15^N_Glu_ enrichment patterns in mycoheterotrophs

Our study integrates four complementary sample sets: (1) fully mycoheterotrophic plants spanning AM‐associated, ECM‐associated, and SAP‐associated taxa; (2) photosynthetic plants with contrasting inferred nutritional modes; (3) co‐occurring autotrophic reference plants that provide local baselines; and (4) fungal material, where available, representing direct nutrient sources. Across all systems, we consistently detected pronounced ^15^N_Phe_ enrichment in mycoheterotrophic species relative to surrounding autotrophs and, where available, fungal partners. Although co‐occurring autotrophic references were unavailable in Fan *et al*. (2025), leaving local nitrogen baselines unresolved, that study also reported strong ^15^N_Phe_ enrichment in *M. humile*.

Using published offsets to approximate fungal baselines (Steffan *et al*., 2015), mycoheterotrophs were enriched in ^15^N_Glu_ and ^15^N_Phe_ by *c*. +2.9‰ and +9.3‰ in AM‐associated taxa, +7.2‰ and +16.7‰ in ECM‐associated taxa, +11.1‰ and +22.1‰ in the partially mycoheterotrophic *P. minor*, and +5.3‰ and +9.7‰ in SAP‐associated taxa relative to inferred fungal baselines, with ^15^N_Phe_ enrichment consistently exceeding ^15^N_Glu_ enrichment. This inversion yields deceptively low TP estimates when standard equations are applied (Chikaraishi *et al*., 2010), indicating that the canonical trophic discrimination factor (Δ^15^N_Glu–Phe_ ≈ 7.6‰) is not applicable to mycoheterotrophic systems. The saprotroph‐associated mycoheterotroph *G. pubilabiata* exhibited minimal ^15^N_Glu_ enrichment but strong ^15^N_Phe_ enrichment relative to its fungal partner (1.5 ± 1.9‰ and 16.3 ± 3.7‰, respectively). Likewise, the AM‐associated *B. cryptopetala* showed clear ^15^N_Phe_ enrichment (11.7 ± 0.5‰) but no ^15^N_Glu_ enrichment (−0.1 ± 0.6‰) in fungus‐free stalks relative to fungus‐containing roots. Although the roots contained mixed plant and fungal tissues, and organ‐level variation in δ^15^N_AAs_ profiles (Takizawa *et al*., 2017) may also contribute to the contrast between roots and floral stalks, these values broadly match the enrichment ranges estimated for AM‐associated taxa relative to fungi.

Overall, our δ^15^N_Glu_ and δ^15^N_Phe_ analyses indicate that mycoheterotrophs exhibit δ^15^N_AAs_ patterns distinct from those of autotrophs, which primarily assimilate inorganic nitrogen. Consistent with previous studies (Courty *et al*., 2011; Gomes *et al*., 2020), bulk δ^13^C and δ^15^N analyses also show that mycoheterotrophs have higher δ^13^C and δ^15^N values than co‐occurring autotrophic plants, although the magnitude of enrichment varies among fungal species and fungal guilds. These isotopic signatures are consistent with the transfer of fungal‐derived organic nutrients. Nevertheless, despite the broad taxonomic coverage, sampling remains limited with respect to within‐plant organ‐level variation and fungal partners. Expanded sampling will be necessary to evaluate the robustness of the patterns documented here.

### Plausible mechanisms underlying ^15^N_Phe_ and ^15^N_Glu_ enrichment patterns

**Fig. 5:**
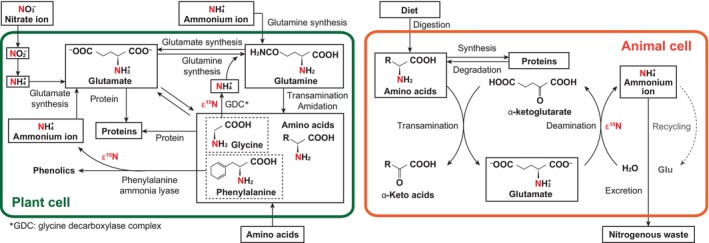
Schematic representation of major nitrogen assimilation and metabolic pathways in plant and animal cells, adapted and modified from Ramirez *et al*. (2021) and Ohkouchi (2023). Regions outlined in green and orange indicate the interiors of plant and animal cells, respectively, whereas areas outside these outlines represent extracellular nitrogen sources and fluxes. In the plant panel, nitrate, ammonium, and amino acids are shown as external nitrogen sources. In autotrophic plants, nitrate and ammonium are the principal inorganic nitrogen sources, whereas fully mycoheterotrophic plants obtain most of their nitrogen through mycorrhizal fungi as organic nitrogen compounds such as amino acids. In the animal panel, dietary nitrogen enters the intracellular amino acid pool after digestion. Arrows indicate uptake, interconversion, synthesis, degradation, excretion, or recycling of nitrogen‐containing compounds, as appropriate. The term ‘amino acids’ denotes the bulk amino acid pool; in plants, phenylalanine and glycine are shown separately because they participate in reactions that release ammoniacal nitrogen (NH_3_/NH_4_^+^). Processes expected to exhibit large nitrogen isotope fractionation are marked with ε^15^N. The gray dashed arrow in the animal panel denotes ammonium recycling; in some taxa, including birds, such recycling may reduce the apparent net nitrogen isotope discrimination expressed at the tissue or organismal level (Wada *et al*., 2025).

We interpret the combination of strong ^15^N_Phe_ enrichment and modest ^15^N_Glu_ enrichment in mycoheterotrophic plants relative to their fungal partners as a consequence of plant nitrogen metabolism (Fig. 5). In contrast to animals (Chikaraishi *et al*., 2009, 2010; McMahon & McCarthy, 2016), in which net ^15^N_Glu_ enrichment can arise through repeated deamination coupled with selective nitrogen excretion, plants typically conserve nitrogen through efficient internal recycling and remobilization (Elser *et al*., 2000; Xu *et al*., 2012). Glu functions as a central hub of nitrogen assimilation and redistribution, and ammonium released during amino acid catabolism is rapidly reassimilated via the glutamine synthetase/glutamate synthase (GS/GOGAT) pathway (Masclaux‐Daubresse *et al*., 2006; Xu *et al*., 2012; Hildebrandt *et al*., 2015). Under conditions of minimal external nitrogen loss and strong internal recovery, net ^15^N_Glu_ enrichment relative to nitrogen inputs is therefore expected to be dampened (Bautista *et al*., 2025; Wada *et al*., 2025).

By contrast, unusually strong ^15^N_Phe_ enrichment likely reflects isotopic fractionation associated with PAL‐mediated deamination during lignin synthesis and broader phenylpropanoid metabolism (Naito *et al*., 2016; Kendall *et al*., 2019). PAL catalyzes the elimination of ammonia from Phe, and kinetic isotope effects preferentially remove ^14^N, thereby leaving the residual Phe pool enriched in ^15^N. Importantly, the amino groups released by this reaction can be rapidly reassimilated into the Glu pool through the GS/GOGAT pathway and then redistributed widely across amino acid metabolism, rather than being returned quantitatively to the same Phe pool (Singh *et al*., 1998). Such broad redistribution would constrain net ^15^N_Glu_ enrichment while allowing ^15^N_Phe_ enrichment.

This interpretation is consistent with broader observations that vascular plants, in which lignin biosynthesis is more pronounced, often show higher δ^15^N_Phe_ values than many algal primary producers (Ramirez *et al*., 2021). However, in autotrophs, Phe is synthesized *de novo* from inorganic nitrogen, and no dietary Phe baseline exists. In mycoheterotrophs, fungal tissues provide an amino acid baseline, including Phe, against which plant Phe can be compared. This allows quantification of plant metabolic fractionation expressed as ^15^N_Phe_ enrichment relative to fungal partners and enables a direct field‐based comparison of plant δ^15^N_Phe_ values with those of nutrient sources. Additionally, variation in ^15^N_Phe_ enrichment among mycoheterotrophic plants likely reflects the balance between fungal Phe supply and diversion of Phe into the phenylpropanoid pathway via PAL. When PAL‐driven flux substantially depletes the available Phe pool, residual Phe should become more ^15^N‐enriched, whereas high Phe availability relative to demand should result in weaker ^15^N_Phe_ enrichment (Kendall *et al*., 2019).

The degree of ^15^N_Phe_ and ^15^N_Glu_ enrichment should also depend on the proportion of inorganic soil nitrogen in plant nitrogen budgets. If mycoheterotrophs obtain most of their nitrogen directly from soil as inorganic forms, primarily nitrate and ammonium, through their roots, ^15^N_Phe_ and ^15^N_Glu_ enrichment relative to autotrophs should be minimal. Consistent with this expectation, AM‐associated mycoheterotrophs, which likely use both fungus‐derived organic nitrogen and soil inorganic nitrogen (Gomes *et al*., 2020), tended to show smaller ^15^N_Phe_ and ^15^N_Glu_ enrichment. Our results also indicate that stronger ^15^N_AAs_ enrichment is associated with greater bulk ^15^N enrichment. Notably, the partially mycoheterotrophic *P. minor* showed the highest enrichment of both ^15^N_AAs_ and bulk ^15^N relative to inferred fungal baselines, consistent with a substantial contribution of fungus‐derived organic nitrogen.

Overall, pronounced ^15^N_Phe_ enrichment accompanied by modest ^15^N_Glu_ enrichment likely reflects plant‐specific nitrogen recycling and metabolic routing. Similar ^15^N_AAs_ enrichment patterns may therefore occur in other heterotrophic plants, including carnivorous and parasitic taxa. Although Fan *et al*. (2025) argued that existing Glu‐Phe‐based trophic‐position frameworks can be applied to carnivorous plants, their estimates were derived solely from Glu‐Phe differences and did not include prey δ^15^N_AAs_ data. Direct comparisons of δ^15^N_AAs_ values between carnivorous plants and their prey will be essential in future studies. Moreover, because parasitic plants have not yet been evaluated using δ^15^N_AAs_ analyses, further work is needed to determine whether they also exhibit weak ^15^N_Glu_ enrichment and strong ^15^N_Phe_ enrichment relative to their nutrient sources.

### ^15^N_Phe_ enrichment as an indicator of fungal‐derived organic nutrient acquisition

Although the mechanisms underlying exceptional ^15^N_Phe_ enrichment are not yet fully resolved, consistent ^15^N_Phe_ enrichment relative to nearby autotrophs provides a promising diagnostic signal of fungal nutrient acquisition when bulk isotope metrics are ambiguous. For example, *T. camschatcense*, regarded as autotrophic despite elevated δ^13^C values (Murata‐Kato *et al*., 2022), showed no ^15^N_Phe_ enrichment, suggesting that this approach can reduce false positives. By contrast, the rhizoctonia‐associated orchid *G. velutina*, inferred to be partially mycoheterotrophic based on the occurrence of albino individuals, exhibited some ^15^N_Phe_ enrichment despite equivocal δ^13^C evidence (Suetsugu *et al*., 2019), suggesting that this method may also reduce false negatives. Because the magnitude and prevalence of carbon transfer through mycorrhizal networks remain central questions (Martin & van der Heijden, 2024; Merckx *et al*., 2024), consistent ^15^N_Phe_ enrichment relative to autotrophic references and, where possible, fungal partners may serve as a qualitative indicator of fungal nutrient acquisition when bulk isotope metrics are inconclusive.

Nevertheless, δ^15^N_Phe_ is not a universal marker of carbon acquisition. δ^15^N_Phe_ primarily reflects nitrogen source and processing, and any inference about carbon acquisition is indirect and depends on coupling between fungal carbon and nitrogen transfer, which is not demonstrated here. Because nitrogen and carbon can follow distinct pathways, ^15^N_Phe_ enrichment should be interpreted alongside complementary evidence, including bulk isotope metrics relevant to mycoheterotrophy such as δ^2^H, δ^13^C, and δ^15^N, together with nitrogen concentrations and other stoichiometric indicators (Gebauer & Meyer, 2003; Hynson *et al*., 2013; Gebauer *et al*., 2016). Notably, δ^15^N_Phe_ did not necessarily increase monotonically with the degree of mycoheterotrophy, as the partially mycoheterotrophic *Platanthera minor* showed stronger ^15^N_Phe_ enrichment than most fully mycoheterotrophic taxa. Broader sampling across partially mycoheterotrophic species will be needed to define the scope and limitations of this approach.

Moreover, the two proposed processes underlying the distinctive ^15^N_AAs_ enrichment pattern, namely nitrogen recycling that suppresses ^15^N_Glu_ enrichment and PAL‐mediated deamination of Phe at the entry point to phenylpropanoid biosynthesis, which could enrich the residual Phe pool in ^15^N, remain hypothetical. Accordingly, ^15^N tracer experiments are required to clarify ^15^N_AAs_ enrichment in mycoheterotrophic plants, ideally using compartmentalized culture systems that restrict labeled substrates to fungal compartments (Bougoure *et al*., 2010). For the first prediction, fungi could be pulsed with ^15^N‐glycine and incorporation into plant Gln, Glu, and ammonium pools could then be tracked over time. Rapid ^15^N incorporation into Gln, minimal external ammonium accumulation, and strong responses to GS/GOGAT inhibition would support efficient plant‐side ammonium reassimilation through the GS/GOGAT pathway (Martin *et al*., 1983; Lea & Miflin, 2003). For the second prediction, transient PAL inhibition with 2‐aminoindan‐2‐phosphonic acid (AIP), followed by quantification of δ^15^N_Phe_, would test for a PAL‐linked isotope effect. This mechanism predicts a dose‐dependent decrease in ^15^N enrichment of residual Phe when PAL activity is suppressed (Hermes *et al*., 1985; Appert *et al*., 2003; Kendall *et al*., 2019). These experiments would establish a mechanistic link between nitrogen metabolism and δ^15^N_AAs_ patterns in mycoheterotrophic plants.

In summary, mycoheterotrophic plants consistently deviate from animal‐derived Glu‐Phe trophic rules. Their pronounced ^15^N_Phe_ enrichment supports the use of this metric as a complementary diagnostic of fungal organic nutrient acquisition in AM and rhizoctonia‐associated systems, where bulk δ^2^H, δ^13^C, and δ^15^N values can be equivocal, while recognizing that this signal may be attenuated by factors such as substantial inorganic nitrogen uptake in some mycoheterotrophic taxa.

## Competing interests

None declared.

## Author contributions

KS and JM conceived and designed the study. KS collected the samples. CY, JM, KS and IT conducted the laboratory experiments. KS and CY performed the analyses. KS drafted the initial manuscript with input from JM and CY. All authors revised the manuscript and approved the final version for submission.

## Disclaimer

The New Phytologist Foundation remains neutral with regard to jurisdictional claims in maps and in any institutional affiliations.

## Supporting information


**Fig. S1** Sampling locations for each plant species.

**Table S1** Mean (±SD) values of δ^13^C, δ^15^N, ε^13^C, ε^15^N, δ^15^N_Glu_, δ^15^N_Phe_, ε^15^N_Glu_, ε^15^N_Phe_, and estimated trophic position (TP) for fully mycoheterotrophic plants associated with AM, ECM, and SAP fungi; a partially mycoheterotrophic plant associated with ECM; an orchid associated with rhizoctonia; a presumed autotrophic plant with significant ^13^C enrichment; and the co‐occurring autotrophic plants, deadwood, and fungal partners at each study site.
**Table S2** Individual values of δ^13^C, δ^15^N, ε^13^C, ε^15^N, C and N concentrations, δ^15^N_Glu_, δ^15^N_Phe_, ε^15^N_Glu_, ε^15^N_Phe_, and estimated trophic position (TP) for fully mycoheterotrophic plants associated with AM, ECM, and SAP fungi; a partially mycoheterotrophic plant associated with ECM; an orchid associated with rhizoctonia; a presumed autotrophic plant with significant ^13^C enrichment; and the co‐occurring autotrophic plants, deadwood, and fungal partners.
Please note: Wiley is not responsible for the content or functionality of any Supporting Information supplied by the authors. Any queries (other than missing material) should be directed to the *New Phytologist* Central Office.

## Data Availability

All data and code used in this study are available on Figshare at https://figshare.com/s/be0e649b4fbbf02b4a78.
