# Supplementary material for: Phenylalanine 15N enrichment likely indicates fungal‐derived organic nutrient acquisition in mycoheterotrophic plants across fungal guilds
Source: New Phytol. 2026 Apr 29;250(6):3990–4001. doi: 10.1111/nph.71154 (PMC13193498; doi:10.1111/nph.71154)
Supplement: Supplementary file 1 — Fig. S1 Sampling locations for each plant species. [file NPH-250-3990-s001.pdf]

New Phytologist Supporting Information

Article title: Phenylalanine ^{15}N enrichment likely indicates fungal-derived organic nutrient acquisition in mycoheterotrophic plants across fungal guilds

Authors: Kenji Suetsugu, Chikage Yoshimizu, Jun Matsubayashi, Ichiro Tayasu

Article acceptance date: 19 March 2026

The following Supporting Information is available for this article:

Fig. S1. Sampling locations for each plant species.

Table S1. Mean (± SD) values of $\delta^{13}C$, $\delta^{15}N$, $\varepsilon^{13}C$, $\varepsilon^{15}N$, $\delta^{15}N_{Glu}$, $\delta^{15}N_{Phe}$, $\varepsilon^{15}N_{Glu}$, $\varepsilon^{15}N_{Phe}$, and estimated trophic position (TP) for fully mycoheterotrophic plants associated with AM, ECM, and SAP fungi; a partially mycoheterotrophic plant associated with ECM; an orchid associated with rhizoctonia; a presumed autotrophic plant with significant ^{13}C enrichment; and the co-occurring autotrophic plants, deadwood, and fungal partners at each study site (separate file).

Table S2. Individual values of $\delta^{13}C$, $\delta^{15}N$, $\varepsilon^{13}C$, $\varepsilon^{15}N$, C and N concentrations, $\delta^{15}N_{Glu}$, $\delta^{15}N_{Phe}$, $\varepsilon^{15}N_{Glu}$, $\varepsilon^{15}N_{Phe}$, and estimated trophic position (TP) for fully mycoheterotrophic plants associated with AM, ECM, and SAP fungi; a partially mycoheterotrophic plant associated with ECM; an orchid associated with rhizoctonia; a presumed autotrophic plant with significant ^{13}C enrichment; and the co-occurring autotrophic plants, deadwood, and fungal partners (separate file).

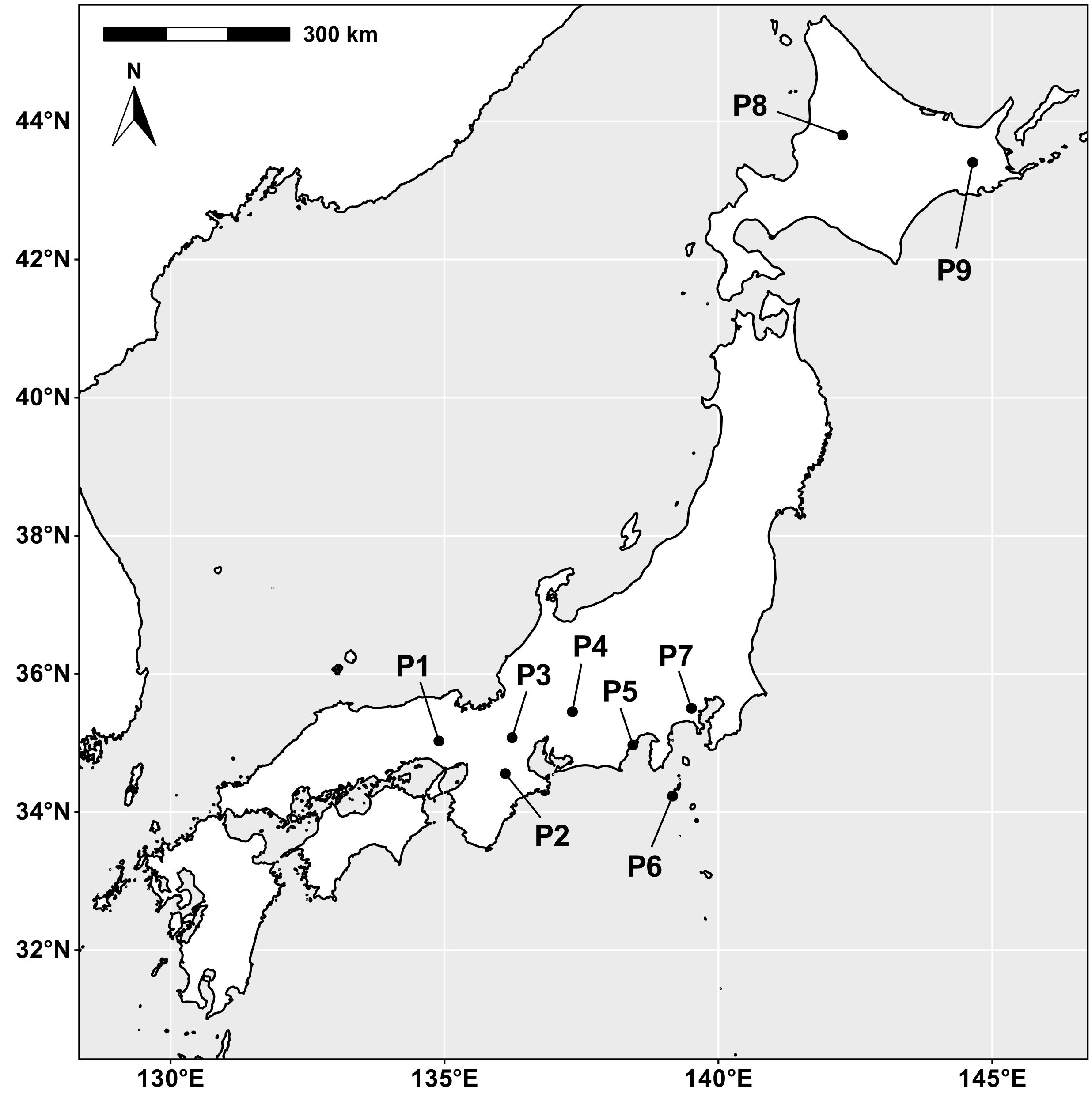

Fig. S1. Sampling locations for each plant species. Closed circles indicate study sites (P1–P9).
